# Authors’ reply

**Published:** 2011

**Authors:** Somenath Kundu, Subhra Mitra, Subhasis Mukherjee, Soumya Das

**Affiliations:** *Department of Respiratory Medicine, IPGMER & SSKM Hospital, Kolkata, India E-mail: susami2@gmail.com*; 1 *Department of Respiratory Medicine, Midnapore Medical College & Hospital, West Midnapore, India*; 2 *Department of Respiratory Medicine, R. G. Kar Medical College & Hospital, Kolkata, India*; 3 *Department of Respiratory Medicine, BS Medical College, Bankura, West Bengal, India*

Sir,

Our thanks to Nikolaos Barbetakis, Christos Asteriou, Christodoulos Tsilikas for sharing their experience with video-assisted thoracoscopic surgery (VATS) in the management of tuberculous empyemas in response to our article “Adult thoracic empyema: A comparative analysis of tuberculous and non-tuberculous etiology in 75 patients”. Fibrinolysis was not included in our study on account of the following. Though there are several studies in favor of the use of fibrinolytics in empyema management, MIST trial (the largest multicentric trial till date on use of fibrinolytics in empyema management) has concluded against the use of fibrinolytics.[1] Also, a Chochrane review conducted in 2004 showed that on the basis of available randomized data, benefits from use of fibrinolytics in empyema were unproven and therefore not recommended.[2] Moreover, overall 58 out of 75 (77.3%) [21 out of 29 i.e. 72.4% of tuberculous empyema; 37 out of 46 i.e. 80.4% non-tuberculous empyema] patients in our study were in stage 3 empyema. We did not keep VATS in our study purview as knowledge about efficacy of VATS in stage 3 empyema is limited, as acknowledged by the letter-writers too.[3] However, considering its lower morbidity and smaller duration of hospital stay compared to open thoracotomy, VATS can surely be a good initial surgical option in selected cases of thoracic empyema (such as incompletely drained para-pneumonic effusions) when conducted in an equipped facility.
